# A novel prognostic model associated with the overall survival in patients with breast cancer based on lipid metabolism‐related long noncoding RNAs

**DOI:** 10.1002/jcla.24384

**Published:** 2022-04-20

**Authors:** Guo‐Jian Shi, Qin Zhou, Qi Zhu, Li Wang, Guo‐Qin Jiang

**Affiliations:** ^1^ Department of General Surgery The Second Affiliated Hospital of Soochow University Suzhou China; ^2^ 105860 Department of Thyroid and Breast Surgery Wuzhong People's Hospital of Suzhou City Suzhou China; ^3^ Department of Thyroid and Breast Surgery The First People's Hospital of Kunshan Kunshan China; ^4^ 569227 Department of Thyroid and Breast Surgery Traditional Chinese Medicine Hospital of Kunshan Kunshan China; ^5^ 569227 Department of Radiotherapy Traditional Chinese Medicine Hospital of Kunshan Kunshan China

**Keywords:** bioinformatic analysis, biomarkers, breast cancer, lipid metabolism, lncRNA

## Abstract

**Background:**

Lipid metabolism is closely related to the occurrence and development of breast cancer. Our purpose was to establish a novel model based on lipid metabolism‐related long noncoding RNAs (lncRNAs) and evaluate the potential clinical value in predicting prognosis for patients suffering from breast cancer.

**Methods:**

RNA data and clinical information for breast cancer were obtained from the cancer genome atlas (TCGA) database. Lipid metabolism‐related lncRNAs were identified via the criteria of correlation coefficient |*R* ^2^| > 0.4 and *p* < 0.001, and prognostic lncRNAs were identified to establish model through Cox regression analysis. The training set and validation set were established to certify the feasibility, and all samples were separated into high‐risk group or low‐risk group. Gene Ontology (GO) and Gene Set Enrichment Analysis (GSEA) were conducted to evaluate the potential biological functions, and the immune infiltration levels were explored through Cibersortx database.

**Results:**

A total of 14 lncRNAs were identified as protective genes (AC022150.4, AC061992.1, AC090948.3, AC092794.1, AC107464.3, AL021707.8, AL451085.2, AL606834.2, FLJ42351, LINC00926, LINC01871, TNFRSF14−AS1, U73166.1 and USP30−AS1) with HRs < 1 while 10 lncRNAs (AC022150.2, AC090948.1, AC243960.1, AL021707.6, ITGB2−AS1, OTUD6B−AS1, SP2−AS1, TOLLIP−AS1, Z68871.1 and ZNF337−AS1) were associated with increased risk with HRs >1. A total of 24 prognostic lncRNAs were selected to construct the model. The patients in low‐risk group were associated with better prognosis in both training set (*p* < 0.001) and validation set (*p* < 0.001). The univariate and multivariate Cox regression analyses revealed that risk score was an independent prognostic factors in both training set (*p* < 0.001) and validation set (*p* < 0.001). GO and GSEA analyses revealed that these lncRNAs were related to metabolism‐related signal pathway and immune cells signal pathway. Risk score was negatively correlated with B cells (*r* = −0.097, *p* = 0.002), NK cells (*r* = −0.097, *p* = 0.002), Plasma cells (*r* = −0.111, *p* = 3.329e‐04), T‐cells CD4 (*r* = −0.064, *p* = 0.039) and T‐cells CD8 (*r* = −0.322, *p* = 2.357e‐26) and positively correlated with Dendritic cells (*r* = 0.077, *p* = 0.013) and Monocytes (*r* = 0.228, *p* = 1.107e‐13).

**Conclusion:**

The prognostic model based on lipid metabolism lncRNAs possessed an important value in survival prediction of breast cancer patients.

## Graphical abstract

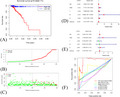

## 1 INTRODUCTION

As the most commonly diagnosed cancer in women, breast cancer may occur in one in eight women during their lifetimes.^1^, ^2^ Although cancer treatment has significantly improved in recent decades, its mortality is still high and accounts for approximately 6.4% of mortality rate.^3^ In recent decades, metabolic changes have been widely observed in a variety of cancer cells.^4^ Due to the consistent change of nutrients in the tumor microenvironment, cancer cells maintain rapid proliferation, survival, migration, invasion and metastasis via lipid metabolism.^5^ Lipid accumulation is recognized as a signature of cancers.^6^ The reduction in lipid accumulation could suppress tumor growth.^7^ Epidemiological studies also proved that fatty acid synthase that plays vital role in lipid metabolism is associated with molecular subtypes and prognosis of breast cancer.^8^, ^9^, ^10^

Long noncoding RNAs (lncRNAs) were defined as a type of RNA more than 200 nucleotides in length without capacity to encode protein. LncRNAs participate in many significant biological processes and are closely related to breast cancer diagnosis and prognosis.^11^, ^12^ However, the mechanism of lncRNAs in transcription is still poorly understood. Our analysis was conducted to identify whether lipid metabolism related to lncRNAs could predict prognosis in breast cancer accurately.

## 2 MATERIALS AND METHODS

### 2.1 Gene expression and clinical information of breast cancer patients

The RNA‐seq data and corresponding clinical information of 1053 breast cancer tissues and 111 normal tissues were downloaded from the TCGA database (http://www.cancergenome.nih.gov/). The data with complete clinical information were retained.

### 2.2 Identifying lipid metabolism‐related genes and related lncRNAs

We identified 146 lipid metabolism‐related genes from gene set “KEGG_GLYCEROLIPID_METABOLISM”, “KEGG_GLYCEROPHOSPHOLIPID_METABOLISM”, “SPHINGOLIPID_METABOLISM” and “ETHER_LIPID_METABOLISM” in Gene Set Enrichment Analysis (GSEA) database (https://www.gsea‐msigdb.org/gsea/index.jsp). Pearson's correlation coefficient was calculated via R v4.0.2. (http://www.r‐project.org/). If the square of correlation coefficient |*R* ^2^| > 0.4 and *p* < 0.001, the lncRNAs were considered to be related genes.

### 2.3 Identifying prognostic lncRNAs

“Survival package” was used to identify prognostic lncRNAs via Kaplan–Meier test. Step Function was applied to narrow down prognostic genes. Sankey diagram and co‐expression network between 24 lncRNAs and 19 mRNAs were constructed via R v4.0.2. and Cytoscape software 3.8.0.

### 2.4 Constructing prognostic model

All samples were separated into training set and validation set randomly. The risk score of each prognostic lncRNAs was calculated to construct the predictive prognostic model. According to previous article, predictive prognostic model was constructed.^13^ All patients were separated into two groups based on the risk score. Kaplan–Meier plot, survival status and prognostic index distribution were drawn to compare the survival differences.

### 2.5 Evaluating signature of clinicopathological variables

Clinicopathological variables (primary tumor status, lymph node status, age and stage) were associated with the prognosis of breast cancer. Clinicopathological variables and risk score of prognostic model were compared through Cox univariate and multivariate analyses. The receiver operating characteristic curve (ROC) plot was drawn to evaluate the accuracy of our model in predicting prognosis of patients.

### 2.6 Gene Ontology and GSEA analyses

“Limma package” was used to identify the differentially expressed genes with the cut‐off criteria of false discovery rate (FDR) < 0.05 and |fold change (FC)| > 2. Differentially expressed lncRNAs were identified to perform Gene Ontology (GO) analysis. GSEA was performed 1000 times to explore the potential functions by using “c2.cp.kegg.v7.2.symbols.gmt” as gene sets database. The p value and normalized enrichment score (NES) were applied to evaluate the potential pathways.

### 2.7 Evaluating the tumor‐infiltrating immune cells

The information of tumor‐infiltrating immune cells was obtained from the CIBERSORTx database^14^ (https://cibersortx.stanford.edu/) that contains the proportion of 22 immune cells in each sample. The proportions of 22 immune cells were compared between high‐risk group and low‐risk group.

## 3 RESULTS

### 3.1 Identification of lipid metabolism related to lncRNAs and prognostic genes

**FIGURE 1:**
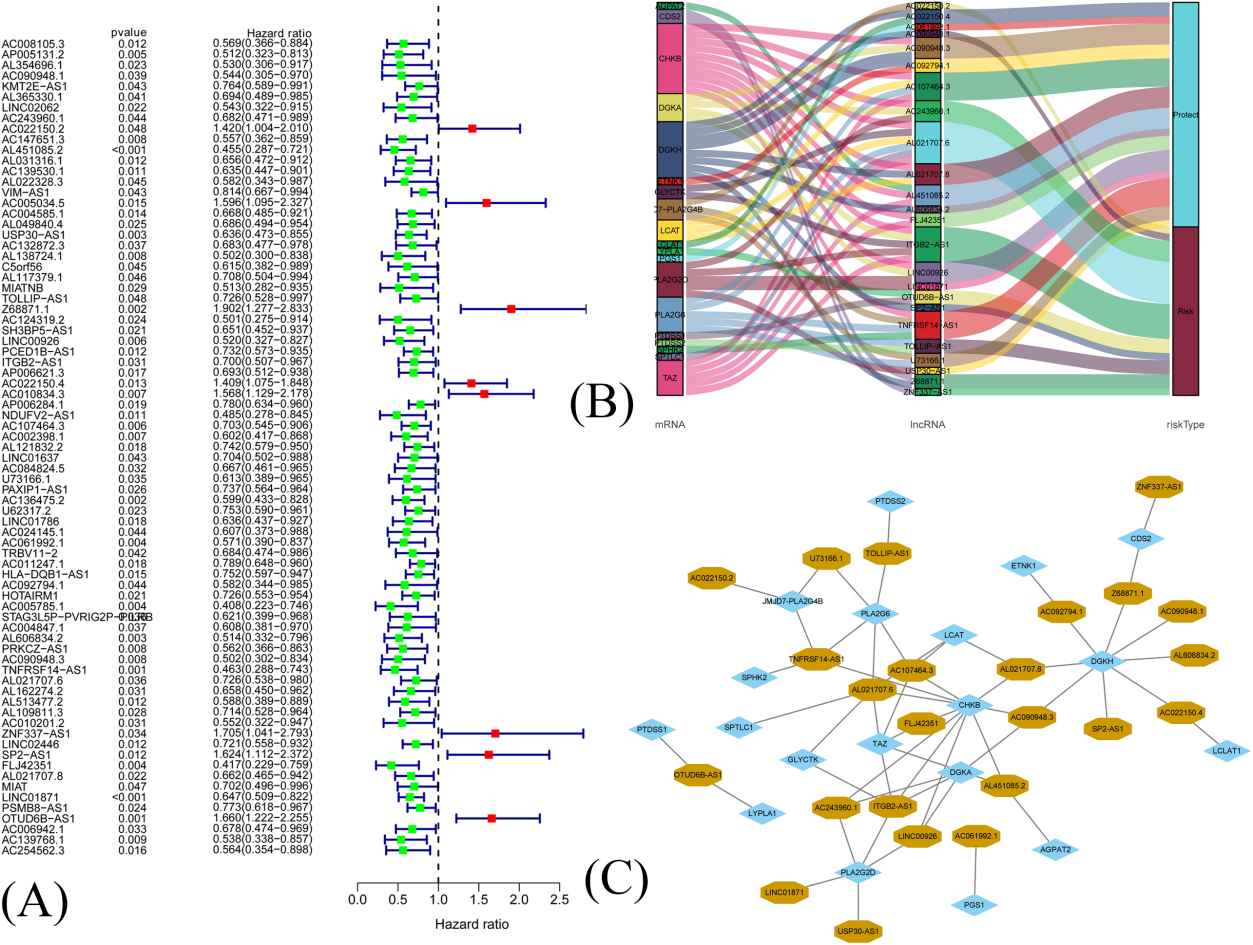
(A) The forest plot of 77 prognostic long noncoding RNAs (lncRNAs) associated with overall survival. (B) The Sankey diagram of 24 lncRNAs and 19 mRNAs. (C) The Coexpression network of 24 lncRNAs and 19 mRNAs

A total of 14,142 lncRNAs were included in TCGA database, and 728 lipid metabolism related to lncRNAs were eligible for selection criteria (|*R* ^2^| > 0.4 and *p* < 0.001). There were 1053 breast cancer samples in TCGA database, and 77 prognostic lncRNAs associated with overall survival (*p* < 0.05, Figure 1A) were identified. Totally, 24 prognostic lncRNAs were narrowed down via Step Function. Among 24 lncRNAs, 14 lncRNAs were associated with better outcome, while 10 lncRNAs were associated with worse outcome (Figure 1B). A co‐expression network was constructed in Figure 1C.

### 3.2 Constructing prognostic model

**FIGURE 2:**
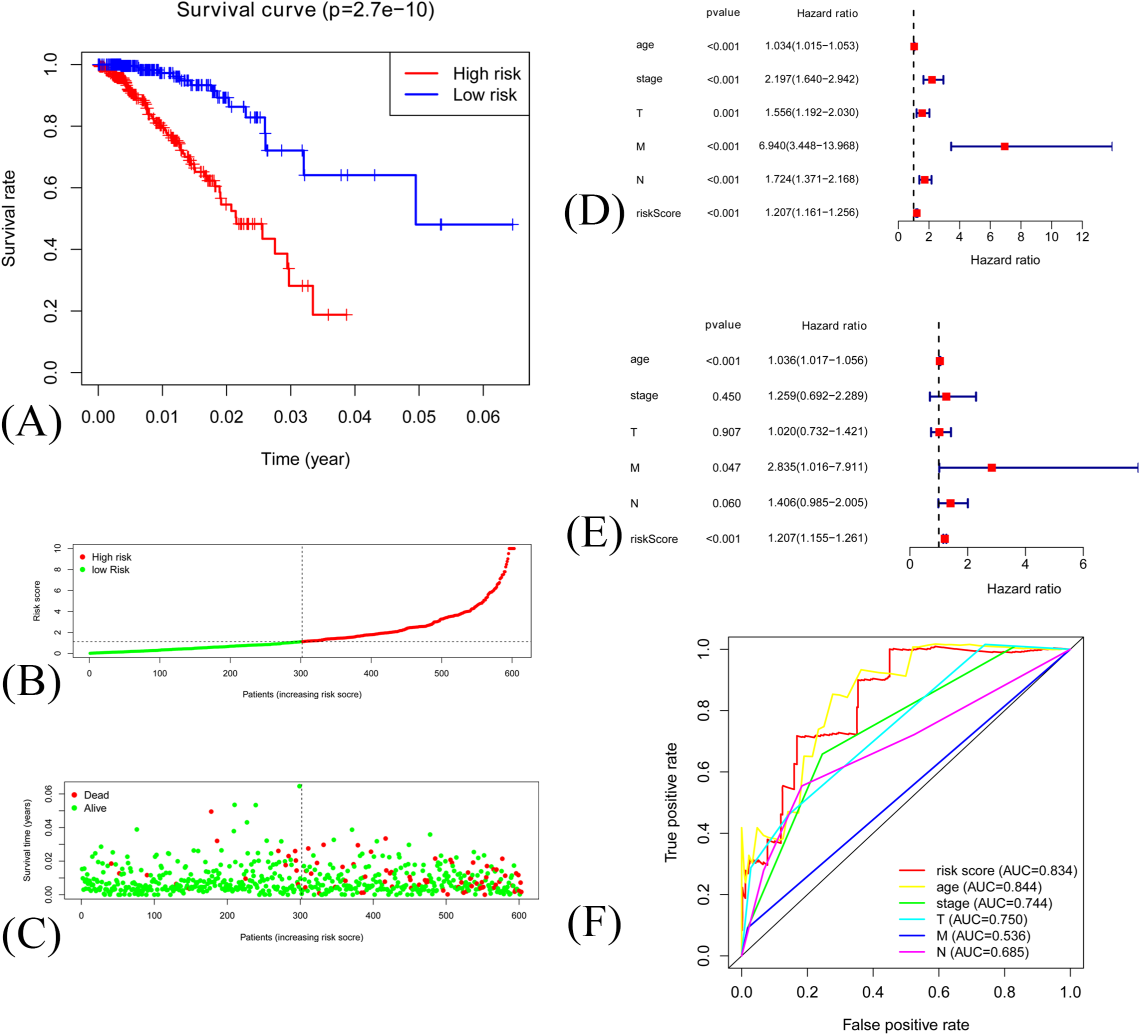
(A) Kaplan–Meier curve of samples in high‐risk group and low‐risk groups in training set. (B) Distribution of risk score in training set. (C) The relationship between survival status and risk score in training set. (D) Forest plot of Cox univariate analysis in training set. (E) Forest plot of Cox multivariate analysis in training set. (F) ROC curve of risk score and clinical features in training set

**FIGURE 3:**
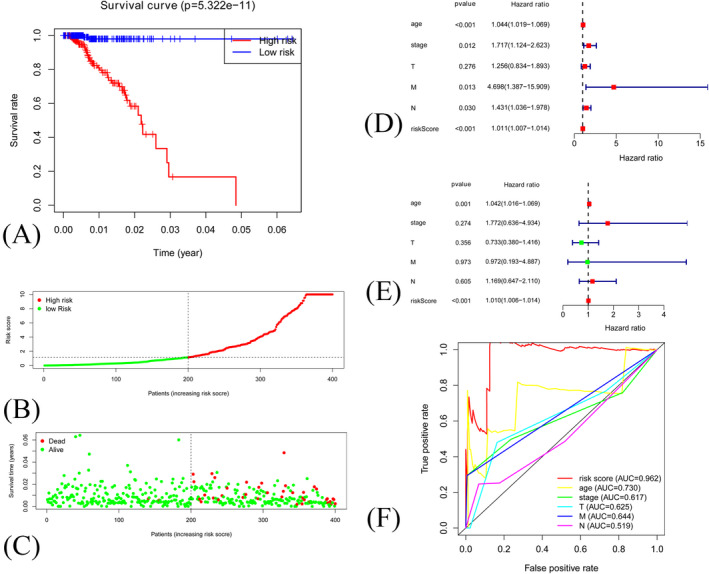
(A) Kaplan–Meier curve of samples in high‐risk group and low‐risk groups in validation set. (B) Distribution of risk score in validation set. (C) The relationship between survival status and risk score in validation set. (D) Forest plot of Cox univariate analysis in validation set. (E) Forest plot of Cox multivariate analysis in validation set. (F) ROC curve of risk score and clinical features in validation set

All samples were divided into training set and validation set randomly at a 3:2 ratio. The characteristics of training and validation set were attached in Appendix S1. Each prognostic gene attains a score, and the risk score of each sample was calculated via the formula. Finally, according to the risk score, each sample was divided into high‐risk group or low‐risk group. High‐risk patients were associated with worse prognosis in both training set (*p* < 0.001, Figure 2A) and validation set (*p* < 0.001, Figure 3A). Survival status and prognostic index distribution were similar in both training set (Figure 2B,C) and validation set (Figure 3B,C). The univariate and multivariate cox‐regression analyses were performed to evaluate whether risk score was an independent prognostic factor for breast cancer. The univariate and multivariate regression revealed that risk score (*p* < 0.001) was independent prognostic factor in both training set (Figure 2D,E) and validation set (Figure 3D,E). Multi‐parameter ROC curves revealed that AUC values for risk score in training set (Figure 2F) and validation set (Figure 3F) were 0.834 and 0.962.

### 3.3 GO and GSEA analyses

**FIGURE 4:**
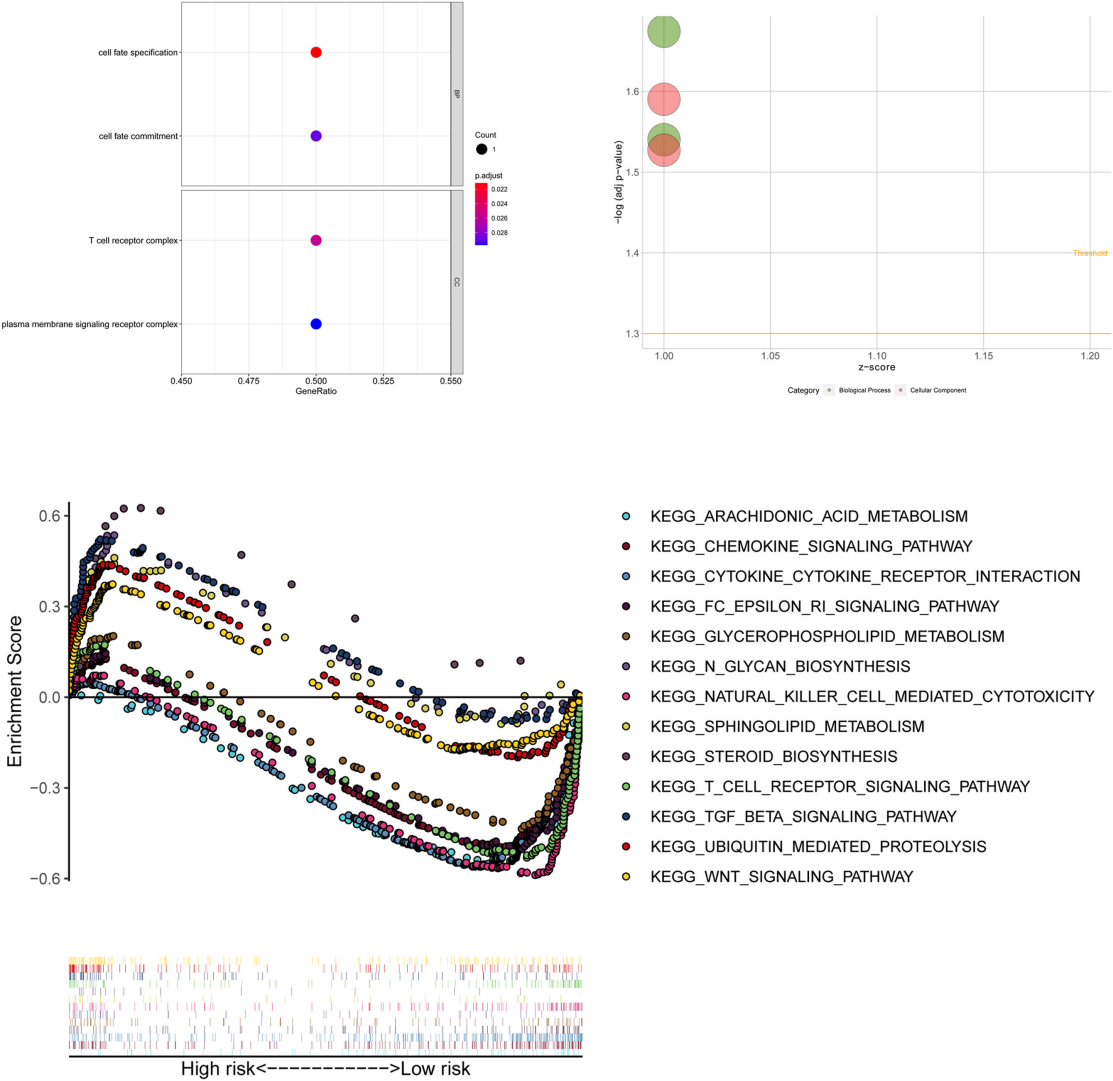
Gene Ontology and gene set enrichment analyses

The enrichment analysis of GO revealed that these lncRNAs were related to cell fate specification, cell fate commitment, T‐cell receptor complex and plasma membrane signaling receptor complex. We selected significantly enriched signaling pathways based on their NES and nominal (NOM) *p* value. The GSEA analysis exhibited significant enrichments in metabolism‐related signal pathway and immune cells signal pathway (Figure 4; Appendix S2).

### 3.4 The infiltrating status of immune cells

**FIGURE 5:**
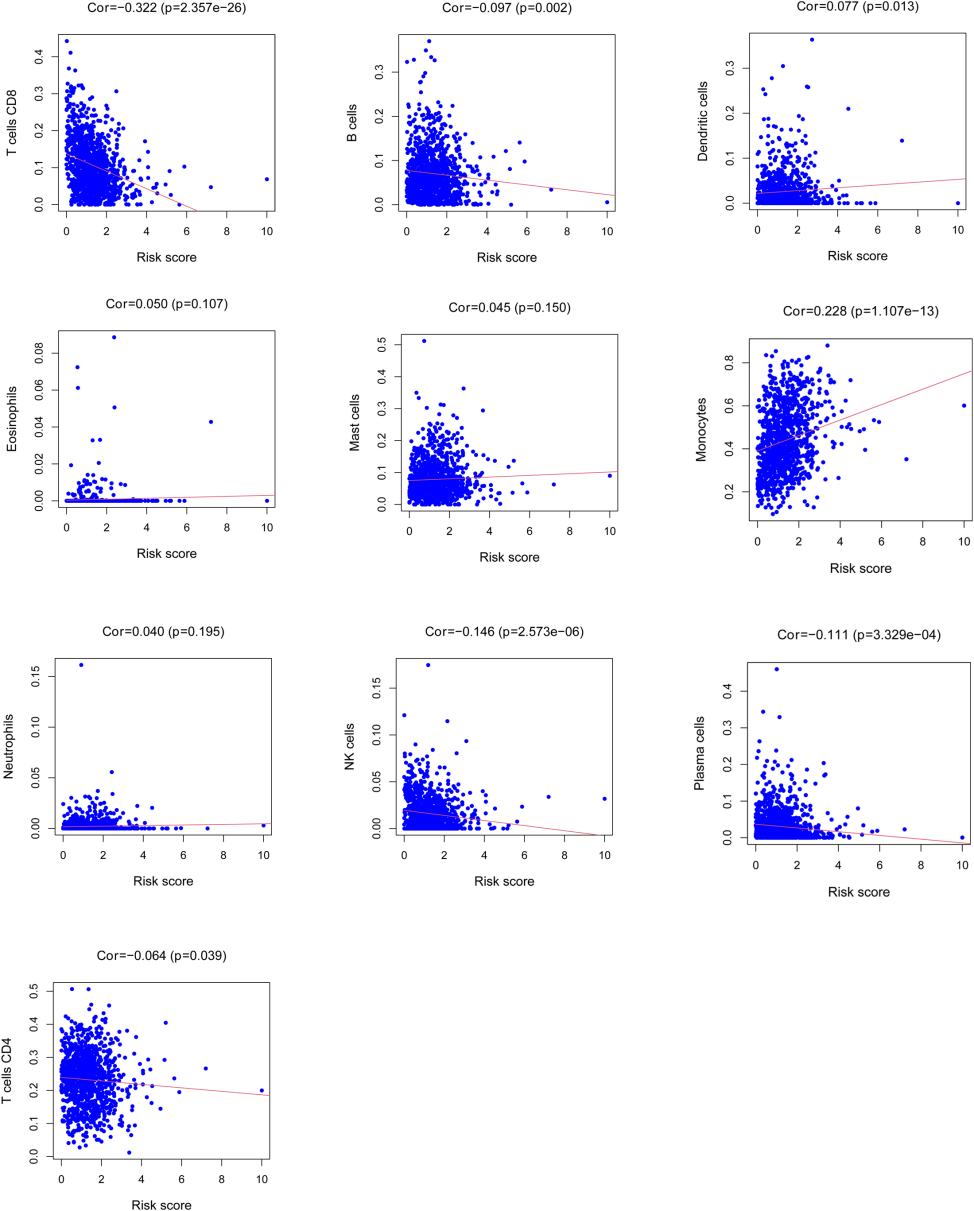
The relationship between risk score and immune infiltration levels

We found that the risk score was negatively correlated with B cells (*r* = −0.097, *p* = 0.002), NK cells (*r* = −0.097, *p* = 0.002), Plasma cells (*r* = −0.111, *p* = 3.329e‐04), T‐cells CD4 (*r* = −0.064, *p* = 0.039) and T‐cells CD8 (*r* = −0.322, *p* = 2.357e‐26) and positively correlated with Dendritic cells (*r* = 0.077, *p* = 0.013) and Monocytes (*r* = 0.228, *p* = 1.107e‐13) via the CIBERSORTx database (Figure 5).

## 4 DISCUSSION

In this study, a novel prognostic model was identified based on lipid metabolism‐related genes. First, 77 prognostic lncRNAs were identified, and narrowed down to 24 genes via Step Function. The risk score was calculated to divide each sample into high‐risk group or low‐risk group on the basis of the prognostic genes. To verify the accuracy and feasibility, all samples were separated into a training set and a validating set. It was observed that patients in high‐risk group were associated with worse prognosis in both training set and validating set. On the other hand, it was found that risk score may be an independent prognostic factors in both training set and validating set. The product of lipid metabolism that secreted into the microenvironment impacts the infiltrating immune cell. Consequently, the status of infiltrating immune cells was analyzed via CIBERSORTx database. It was observed that the risk score may affect the status of B cells, NK cells, Plasma cells, T‐cells CD4, T‐cells CD8, Dendritic cells and Monocytes. GO and GSEA analyses were performed to explore the biological function. It was found that genes included in our model were associated with cell fate specification, cell fate commitment, T‐cell receptor complex and plasma membrane signaling receptor complex. GSEA analysis exhibited a significant enrichment in metabolism‐related signal pathway, immune cells signal pathway and cancer‐related signal pathway.

To our knowledge, AC022150.4, AC107464.3, AL021707.8, AL451085.2, AL606834.2, FLJ42351, TOLLIP‐AS1 and U73166.1 have not been reported. In addition, the mechanism and biological functions of AC022150.2, AC061992.1, AC090948.1, AC090948.3, AC092794.1, AC243960.1, AL021707.6, LINC00926, SP2‐AS1, TNFRSF14‐AS1 and Z68871.1 have not been previously investigated in cancer. Previous studies indicated that ITGB2‐AS1 could promote progression, migration and invasion in many types of cancers, including pancreatic ductal adenocarcinoma, renal cell carcinoma, osteosarcoma and breast cancer.^15^, ^16^, ^17^, ^18^ Chu et al. proved that FOXO3A/LINC00926/PGK1 is a critical axis to regulate breast cancer growth and progression. In this axis, LINC00926 inhibits proliferation, migration and invasion in breast cancer via PGK1‐mediated Warburg effect.^19^ These findings are consistent with our analysis. OTUD6B‐AS1 may act different roles in different cancers. OTUD6B‐AS1 suppresses viability, migration and invasion in thyroid carcinomas, colorectal cancer cell and renal cell carcinoma.^20^, ^21^, ^22^, ^23^ On the other hand, OTUD6B‐AS1 promotes hepatocellular carcinoma cells proliferation and invasion and induces chemoresistance in breast cancer cell and cervical cancer cell.^24^, ^25^, ^26^ How to make a wide use of OTUD6B‐AS1 is worth exploring and may provide a novel strategy to cancer treatment. The diversity of the composition of immune cell may promote tumor development and influence the response to therapy.^27^ The infiltrating status of immune cells analysis revealed that B cells, NK cells, Plasma cells, T‐cells CD4, T‐cells CD8, Dendritic cells and Monocytes were associated with the risk score of our model. Among these immune cells, CD8^+^ T cells were most relevant to the risk score. CD8^+^ T cells plays critical portion in anti‐tumor mechanism. The low level of CD8 T‐cell infiltration status predicts rapid progression and inefficient response to immunotherapy.^28^ Yang et al. indicated that the inhibition of ACAT1 (known as a key enzyme in lipid metabolism) contributes to the increase in plasma membrane, which leads to the proliferation of CD8^+^ T cells via enhancing T‐cell receptor aggregation and signal transduction.^29^ In our results, it was obvious that the risk score was negatively correlated with CD8^+^ T cells. GSEA also revealed that risk score was down‐regulated in T‐cell receptor signaling pathway, which may acquire a better understanding of immune cells functions in lipid metabolism signaling pathway.

There are several limitations in our study. All breast cancer information was obtained from the TCGA database, and the patients were primarily Americans. Breast cancer patients from other regions further require confirmation with additional evidence. Inevitable bias exists in the study, because the validation set was also form TCGA database.

## 5 CONCLUSION

In summary, a novel prognostic model that could predict the prognosis of breast cancer patients based on 24 lipid metabolism related to lncRNAs was identified. This prognostic model not only guides the occurrence of breast cancer but also could provide evidence of the response to immunotherapy.

## CONFLICT OF INTEREST

None declared.

## AUTHOR CONTRIBUTIONS

Conceptualization: Guojian Shi. Data curation: Qin Zhou. Formal analysis: Qi Zhu. Investigation: Qin Zhou, Li Wang. Methodology: Guoqin Jiang. Project administration: Qi Zhu. Supervision: Guojian Shi, Guoqin Jiang. Writing – original draft: Guojian Shi. Writing – review & editing: Li Wang, Guoqin Jiang.

## Supporting information

Appendix S1

Appendix S2

## Data Availability

All data analyzed in this study could be obtained from TCGA and CIBERSORTx database.
